# Copy-Pasting in Patients’ Electronic Medical Records (EMRs): Use Judiciously and With Caution

**DOI:** 10.7759/cureus.40486

**Published:** 2023-06-15

**Authors:** Bassim Al Bahrani, Itrat Medhi

**Affiliations:** 1 Medical Oncology, The Royal Hospital, Muscat, OMN; 2 Medical Oncology, Gulf International Cancer Center, Abu Dhabi, ARE

**Keywords:** patient safety, royal hospital muscat, copy paste, emr, electronic records

## Abstract

An electronic medical record (EMR) is an electronic, comprehensive, and up-to-date compilation of a patient's medical history and information stored in a secure digital format. It provides real-time access to patient data, enabling healthcare providers to make informed decisions quickly and accurately. EMR systems streamline a patient's healthcare journey and enable shared care across the medical practice. By providing a comprehensive view of a patient's medical history, EMRs can be invaluable tools for physicians and healthcare providers, allowing them to collaborate more effectively and provide better care. Additionally, EMRs can help reduce paperwork, improve accuracy, and increase efficiency, ultimately leading to improved patient outcomes. The true potential of EMR systems can be realized when they are used in conjunction with evidence-based medicine methodologies, quality improvement initiatives, and team-based care. This combination of technologies and practices can revolutionize healthcare delivery, improving patient outcomes, greater efficiency, and cost savings.

"Copy-pasting" is an essential feature of EMR systems, with physicians relying on it for up to 35.7% of their workflow. By leveraging the copy-pasting feature of their EMR system, physicians can ensure that their data capture is accurate and timely, leading to better patient care. Copy-pasting can be a valuable tool for physicians, saving time and allowing them to focus on practical clinical issues. However, it is essential to note that while most clinicians copy-paste, 25% of them believe it can lead to a high frequency of medical errors, with the potential for a significant number of errors being attributed to this practice. Therefore, physicians must exercise caution when copy-pasting and take the necessary steps to ensure accuracy and reduce the risk of errors.

Copy-pasting can cause severe adverse patient events by introducing new inaccuracies, rapidly spreading inaccurate or outdated information, leading to discordant notes, and creating long notes that mask essential clinical information. Despite these risks, copy-pasting has become widely used in EMRs. Additionally, copy-pasting can reduce the time spent on documentation, allowing healthcare providers to focus more on patient care. Inappropriate copy-pasting can have serious consequences, such as compromising data integrity, endangering patient safety, increasing costs, and even leading to fraudulent malpractice claims.

In conclusion, copy-pasting can be helpful for healthcare professionals, but it must be used cautiously. Proper education and safeguards should be implemented to ensure accuracy and up-to-date patient data. Additionally, healthcare professionals should be aware of the legal implications of copy-pasting, as it may be considered a form of medical malpractice. With the proper precautions, copy-pasting can be a safe and efficient way to save time and reduce errors in patient records.

## Introduction and background

Medical record history can be traced back to the fifth century B.C. when Hippocrates proposed two critical objectives for medical records: to provide a comprehensive record of a patient's medical history and to serve as a tool for medical research. Hippocrates also recognized the importance of maintaining accurate and up-to-date records and the need for confidentiality and privacy. Since then, medical records have become an essential part of modern healthcare, providing a comprehensive overview of a patient's medical history and helping to ensure the best possible care [1]. Electronic medical records (EMR) systems have revolutionized the way patient information is stored and accessed. These systems provide a comprehensive, patient-centered experience, allowing authorized users, individuals, and groups to access information in real-time securely. EMRs can store a comprehensive range of patient health data, providing a detailed overview of their healthcare journey, including medical history, progress notes, clinical diagnoses, current medications, allergies, lab results, clinical assessments, treatment plans, and radiologic assessments with images. This data can be used to inform decisions about the patient's care, helping to ensure they receive the best possible treatment. By streamlining the patient's healthcare journey, EMR systems enable shared care across authorized providers and other medical practices [2-6].

EMRs have been in clinical practice since 1960 and are now the standard of medical record keeping in current medical practice. They serve as an electronic version of the patient charts in a healthcare service, allowing healthcare providers to trace data over time, identify due appointments, improve coordination within healthcare service, enhance security, and amplify efficiency and overall quality of care within the practice. EMRs are excellent instruments for physicians and healthcare providers in collaboration with other healthcare professionals. The benefits of EMR systems can be extracted when coupled with the transformation of practices based on quality improvement methodologies, team-based care, and evidence-based medicine. Additionally, EMRs can help to reduce medical errors, improve patient safety, and provide better access to patient information. Furthermore, EMRs can help to reduce paperwork, streamline administrative processes, and improve communication between healthcare providers [2,6,7]. They can also provide real-time access to patient data, allowing for faster diagnosis and treatment. Finally, EMRs can help improve patient engagement and satisfaction by providing access to their medical records and allowing them to be more involved in their healthcare.

Doctors are trained to be discreet, cautious, and prudent about adopting new techniques and systems. They rely on solid evidence and documentation rather than a new way of doing things that will benefit them, their patients, and the services provided. Therefore, providing them with the necessary resources and training is essential to ensure they can use the EMRs to their fullest potential. Most healthcare institutes have converted to EMRs to standardize documentation, avoid errors, concisely monitor, and enforce efficiency and better patient outcomes. EMRs have been proven invaluable in improving patient safety, reducing medical errors, and enhancing the overall quality of care. By providing healthcare providers with a comprehensive, up-to-date view of a patient's medical history, EMRs enable them to make more informed decisions and provide better care.

Additionally, EMRs can help streamline administrative processes, reduce paperwork, and improve communication between healthcare providers. Ultimately, using EMRs can help ensure patients receive the highest quality of care possible [2-7]. Additionally, EMRs can reduce costs, improve communication between healthcare providers, and provide better access to patient information. With the right resources and training, doctors can use EMRs to ensure patients receive the best care.

The aim of this study was to analyze extensive electric medical record facilities objectively, with reference to the copy-pasting option in EMR. We looked into the copy-pasting option with the objective of its use, benefit, limitations, and pitfalls.

## Review

The published literature was searched online using the relevant electronic databases PubMed, MedicinePlus, Embase, Proquest, Scopus, Google Scholar, and ResearchGate using the keywords electronic medical records, EMR, copy and paste, and patient safety in different combinations for the last 20 years from 2003 to 2022. The relevant articles were reviewed in detail and relevant information was extracted from these published articles. Articles included were those which were written by clinicians and critically analyzing the role of EMR and copy-pasting. All non-medical articles were excluded.

The advantages of EMRs

EMRs offer numerous advantages over traditional medical record-keeping systems [3,4,6,7]. These include real-time record access, which allows for swift and straightforward retrieval of patient records, reducing the need for manual data entry and improving accuracy. It causes a reduction of paperwork and the need for physical storage space and human resources, resulting in cost savings. There is increased capacity to manage the large volume of data generated accumulated for each patient, allowing for more comprehensive patient care, clinical alerts, and reminders to reduce and prevent medical errors, improving patient safety. It standardized patient assessments, radiology and lab results, etc., allowing for better communication between healthcare providers and improved documentation accuracy. It simplifies and expedites diagnosis and requests investigations, tracks and manages medications in one place, sends medication alerts for interactions, facilitates tracking and implementation of preventive care, improves point-of-care decision-making, and allows for more straightforward and efficient integration of evidence-based clinical guidelines. There is improved caregiver productivity and the ability to treat more patients more effectively, swift access to patient records to prevent errors like double prescribing medications or test requests, and improved physician communication and collaboration. This is cost-effective and time-saving because it allows for instant communication with insurers, hospitals, and referring physicians; the ability to capture external documents and save them, increasing their diversity and scope; and multiple layered security options and access control, ensuring only authorized access to sensitive patient data portals for electronic interaction between the service provider and patient. The records cannot be altered after a predefined time interval or event, leading to improved clinician satisfaction and enhanced patient satisfaction, including reduced turnaround time for responding to messages and medication refill requests, improved patient continuity of care, improved legal and regulatory compliance, improved ability to aggregate data, conduct research, and surveillance, and improved patient engagement and satisfaction. Additionally, EMRs can provide improved access to patient data for research and public health initiatives and improved data sharing between healthcare providers.

Disadvantages of EMRs

There can be some hazards, risks, and drawbacks associated with EMRs, as outlined below [3,6-9]: EMR systems can be expensive and require regular upgrading and financial and human resources investment. Electronic records are more costly than paper records and cost a lot to install and maintain. It may also need onsite technical support and permanently hired technical personnel. There can be interruptions in smooth workflow due to the time required to absorb and understand a new system. Physicians devote more time to EMR sitting in front of a screen, thus, decreasing the time spent physically with the patients, significantly reducing patient satisfaction and physical assessment.

EMRs contain sensitive patient data, making them a prime target for malicious cyber-attacks. Misusing EMRs or not including key cybersecurity features can lead to legal violations and high penalties. It can expose data to hackers, malware, ransomware, phishing attacks, and cloud threat. There can be insufficient encryption and employee insider threats as well. If remote or cloud EMR software does not have a reliable information backup system in place, all sensitive and invaluable data may be at risk of being lost in the event of a technical error or system failure. To ensure the safety of this data, it is essential to have a secure backup system in place that can be used to restore the data in the event of an emergency. Supposedly, EMR data needs to be updated promptly with new information, such as following an examination or after test results. If not done promptly, it can lead to incorrect or incomplete information being obtained by anyone who scrutinizes the EMR. This can have serious consequences, such as incorrect diagnosis, inappropriate treatment, and poor health outcomes.

EMR software providers must regularly update the EMR system and execute maintenance. If the developer fails to provide timely updates, data breaches and legal problems will likely occur. Implementing an EMR system predisposes to several liability concerns. There is an urgent need to ensure that valuable medical data is accurately and securely transferred from paper to electronic records. Failure to do so can lead to errors in treatment, resulting in physicians being held liable for not having access to all the necessary medical data. It may take years of careful planning and implementation to select and assemble an EMR system and ultimately transition from paper records to digital ones, balancing the requirements and the financial resources available. Once you have determined your budget and identified the features you need in an EMR system, ensuring the system is set up and running smoothly is vital. To ensure a successful transition, adequate training must be provided to your staff; this will help them become familiar with the new system and ensure that they can use it effectively.

Maintaining an EMR system can be time-consuming and challenging as frequent updates are necessary to ensure the system remains secure and efficient. Otherwise, the value of your records can regress in terms of accuracy and significance. EMRs can sometimes be inconvenient, requiring access to a computer and a reliable internet connection. In the event of a power outage or computer malfunction, the EMR information can become inaccessible, potentially disrupting patient care. To ensure that patient care is not compromised, a robust EMR system must have an experienced IT (information technology) team available to resolve any technical issues quickly.

Barriers to implementing EMRs

There are numerous barriers to implementing EMRs, which may be monetary, logistic, or user-related [2,3,6,9]. These barriers are outlined as follows:

Financial Resources Available

Implementing EMRs can be prohibitive for many healthcare providers, especially those in rural or underserved areas. Additionally, the cost of training staff and maintaining the system can be a significant burden.

Physician's Acceptance

Healthcare providers have mixed feelings about EMRs. Many reports suggest that introducing EMRs to their practice has increased the workload of physicians, specialists, and nurses, often leading to frustration [3,6]. Despite the good intentions behind EMRs, the additional workload can be overwhelming and challenging to manage.

User Easiness and Convenience

EMRs can be difficult to use and require significant learning time. Additionally, the user interfaces need to be more intuitive and user-friendly, making it difficult for users to access the information they need quickly.

Lack of Standards

There needs to be more standardization across different EMR systems, making it difficult to transfer data between systems. Additionally, different systems may have different data formats, making it difficult to interpret the data.

Privacy and Security Concerns

EMRs contain sensitive patient information, which must be kept secure. This requires robust security measures to be in place, such as encryption and access control.

Legal Issues

Several legal issues must be addressed when implementing EMRs, such as patient consent, data privacy, and data sharing.

Incomplete or Missing Data

EMRs may contain incomplete or missing data, leading to inaccurate diagnoses or treatments.

Unawareness of the Legal Requirements

Healthcare providers may need to learn the legal requirements for implementing EMRs, such as patient consent and data privacy. Additionally, they may need to gain knowledge of which diseases are reportable and how to report them following applicable laws and regulations. EMRs may contain inaccurate, non-interpretable, or inconsistent data from multiple sources, making it difficult to interpret.

Interoperability

EMRs must be able to communicate with other systems in order to share data. This requires robust interoperability standards to be in place. Phenotyping algorithms are powerful tools for accurately identifying cases by combining various types of data and their logical relationships. These algorithms must be accurate and reliable in order to be effective. The algorithms must be regularly updated to ensure accuracy and keep up with medical knowledge changes.

Technical Support

Technical support is essential for the successful implementation of EMRs. This includes training staff, troubleshooting issues, and ongoing maintenance and support.

Copy-pasting option in EMRs

EMRs are gathered and stored on a computer system, allowing physicians to document a patient's clinical status and condition quickly, accurately, and completely. Computer operating systems provide a range of valuable features, including the invaluable ability to copy-paste. This can be a huge time-saver [9-12]. This feature is present in almost all EMR systems, making it easier for healthcare providers to access and use the information.

**Table 1:** Pros and cons of copy-pasting EMR: electronic medical record

PROS	CONS
Fast and quick	Outdated data can be transferred
Time saver	Must not replace original content but supplement it
Easy	Need verification of data accuracy, which is often ignored
Most people practice it	The source of data is often not ascertained and ignored
Almost all EMR systems allow it	Risk of errors and repeating of errors in data
Improved efficiency	Introduce new inaccuracies
Improved consistency	Can compromise patient safety
Ensure completeness of the information	Medico-legal implications
	Discordant notes obscuring data

According to a recent study, physicians typically spend 44% of their time on the computer, 26% on clinical documentation, and 18% typing on computers. This significant amount of time could be better spent providing direct patient care [13]. "Cut-and-paste" refers to cutting text from one location and pasting it into another during manuscript editing or re-writing. Almost all EMR software allows for the seamless transfer of patient data from one part of the record to another. This can be done quickly and easily using the copy-pasting function, ensuring that all relevant information is accurately and securely transferred. Multiple definitions describe copy-pasting, such as copying matching phrases of more than four words or 20 characters between two documents, or a note containing 20% copied text from another document will be considered copying, or 40 identical consecutive words between two documents will be defined as a copy event [8,11-14].

Additionally, copy-pasting can refer to taking text from one document and placing it into another. It is important to note that copy-pasting should not replace the original content but rather supplement it. Furthermore, ensuring that the copied content is accurate and up-to-date is essential. Additionally, it is crucial to ensure that the copied content is appropriately attributed to its source. To meet timeline targets, physicians often trust and rely on the copy-pasting function, which is used by around 35.7% of physicians [13,15]. This function offers several advantages, such as improved efficiency in capturing data, consistency, completeness, and communication. It also helps to reduce the time spent on documentation and administrative tasks, allowing physicians to focus more on practical clinical issues and providing quality patient care. Additionally, it can help to improve accuracy and reduce errors in data entry, resulting in more reliable and up-to-date patient records [14,16]. However, a mistake in copy-pasting is reported to contribute to over 36% of errors [13] and can have severe consequences for patient safety. It can introduce new inaccuracies, propagate inaccurate or outdated information, create discordant notes, and lead to note bloat, which can obscure important clinical information. These errors can potentially lead to adverse patient events, making it essential to reduce the risk of copy-pasting errors. Clinical studies have demonstrated that the frequency of copy-pasting is significantly associated with the likelihood of readmission for the same disease within two weeks [8,13,16,17]. To reduce the risk of errors, it is essential to ensure that the copied content is accurate, up-to-date, and correctly attributed to its source. Additionally, It is essential to have a clear and agreed-upon definition of copy-pasting to ensure accuracy and consistency in its use.

An analysis of 51 published papers revealed that 66-90% of physicians routinely use the copy-pasting option when creating patient notes, generally without modification or editing [14]. Moreover, nearly 78% of them use it most of the time, while an even higher 81% copy-paste notes from other clinicians or prior admissions [8]. Although this practice may seem convenient, it can seriously affect patient care. Copy-pasting can affect the decision of subsequent physicians and may lead to creating long, disorganized notes that do not accurately reflect a patient's current status. It can also distract a reader from essential concerns, thus, leading to misdiagnosis and incorrect treatment. Therefore, it is important to exercise caution while using the copy-pasting option and ensure that the notes are accurate and up-to-date. There is a growing consensus among physicians that the documentation quality has decreased since the introduction of EMRs. Despite this, many clinicians rely solely on these flawed notes for decision-making, making EMRs the primary source of clinical communication. While it is technically achievable to restrict the copy-pasting function within the EMR, clinicians predictably oppose such radical actions due to the potential for an increased workload and decreased efficiency [8]. This inaction to improve the use of a tool acknowledged as unsafe is a source of imperfection in practice [10,12,14,17]. An extensive survey of physicians suggests that 25% believe that copy-pasting leads to a high frequency of medical errors. Due to study limitations, the direct evidence concerning risk to patient safety remains limited.

Based on prevailing evidence, there are four safe practice recommendations to mitigate the risks associated with copy-pasting: provide a means to ensure that the copy-pasted material is readily identifiable, make sure that the source of the copy-pasted material is identifiable instantly, provide staff training and education in the use and safety of copy-pasting, and ensure that copy-pasting practices can be regularly assessed, measured, and monitored.

Despite the apparent risks associated with the copy-pasting practice, only 24% of institutes have a policy to address these issues [8,14,18]. To ensure the safety of all stakeholders, all institutes must implement a copy-pasting policy that adheres to the aforementioned safe practice recommendations. Furthermore, institutes should regularly review and update their copy-pasting policies to remain up-to-date with the latest evidence and best practices.

Despite the many benefits of computing and EMRs, the widespread use of EMRs has led to concerns about the potential dangers of the copy-pasting functionality. In 2012, Hirschtick highlighted the risks associated with copy-pasting [16]. The patient, believed to have a history of pulmonary embolism (PE), received an unnecessary CT scan to rule out a suspected recurrent pulmonary embolus. However, the PE was used in the electronic note to indicate a physical examination, not a pulmonary embolism [9,17]. This case serves as a reminder of the importance of carefully reviewing all medical records before making decisions. This case highlights the importance of understanding the implications of copy-pasting and the need for clinicians to be aware of the potential risks associated with its use. To ensure patient safety, it is essential to implement policies and procedures that limit copy-pasting and ensure that the information is accurate and up-to-date. Clinicians should be encouraged to copy-paste responsibly and to take the time to review and edit the information before it is included in the patient's record.

We as clinicians remain skeptical about copy-pasting and patient safety. A literature review has revealed that over 35% of errors can be attributed to copy-pasting mistakes [10,19]. To address this issue, some professional international associations and organizations have published consensus reports and developed comprehensive materials for education and mentoring [3,20]. Furthermore, some EMR suppliers have developed sophisticated tools for detecting copy-pasted material and the source of the copied content. These tools can accurately pinpoint the origin of each character within a transcript notation, whether it is newly typed, imported from another source, or copy-pasted [18].

However, only a few institutes use these tools to educate physicians to improve their documentation quality. More published data is available about initiatives on documentation improvement addressing the copy-pasting function [21-23]. To ensure that copy-pasting is used appropriately and that patient safety is not compromised, it is essential to supervise its use and give feedback to our trainees and fellows when it is being misused. Documentation review should be incorporated into peer review processes to ensure that copy-pasting is used appropriately and that patient safety is not compromised. Additionally, educational sessions should be provided to the new generation of young clinicians, trainees, and fellows to ensure they understand the appropriate use of copy-paste-review processes to improve communication and patient safety [14].

Furthermore, healthcare organizations should consider implementing regular audits and feedback sessions to ensure that copy-pasting is used appropriately. Additionally, healthcare organizations should consider developing policies and procedures to ensure that copy-pasting is used safely and effectively. These policies and procedures should include guidelines for when copy-pasting should be used, how it should be used, and how to identify and address potential errors.

Discussion

EMRs are an invaluable tool for healthcare providers, providing a comprehensive and detailed record of a patient's medical history. Unlike other electronic files, EMRs capture a wide range of information, including patient demographics, medical history, laboratory data, radiology reports and images, physician opinions, and real-time treatment adjustments. The extensive implementation of EMRs has steered considerable improvement in the transformation of healthcare service delivery [2,13,16]. The benefits of implementing EMRs are numerous, including improved access to patient information, increased quality of patient-centered care, clinical decision support, cost savings, and improved data management for medical research and education. Additionally, EMRs can help reduce medical errors, improve patient outcomes, and provide better continuity of care while streamlining administrative processes and improving communication between healthcare providers [3,6,13].

Copy-pasting, also known as cloning, is a widely used technique in patient EMRs that allows users to quickly and easily transfer information from one source to another. This practice can save time and effort, eliminating the need to re-enter data manually. Additionally, it helps to ensure accuracy, as the copied information is exact and up-to-date. While it can be an efficient note-taking method, it can also be a potentially dangerous practice if not done correctly, leading to medical errors, incorrect transmission of information, or lack of continuity of care [24]. These errors can be especially detrimental in a medical emergency, where the lack of accurate information can lead to delays in treatment and more severe consequences. Moreover, due to the sharing of EMRs among healthcare providers, incorrect information can be quickly spread and become a source of confusion, leading to further errors and misperceptions [25].

Copy-pasting has also been criticized, as it can produce inaccurate records (outdated, incorrect, or incomplete), leading to medical errors, and can be a source of fraud [24]. It can also lead to potential legal liabilities for healthcare professionals, inappropriate invoicing to patients and third-party healthcare payers, and inflated or fraudulent malpractice claims. Additionally, copy-pasting can trigger a range of data integrity issues, such as unreasonably long, poorly organized notes, inaccurate encounter tracking, diagnosis errors caused by false assumptions or attributions, and regulatory concerns about the accuracy and necessity of billed services. These issues can lead to significant financial and legal repercussions and decreased patient safety and quality of care. The legal responsibility of copy-pasting errors is with the person doing copy-pasting. That is why he must ensure safe practice, avoid errors, and do it with responsibility.

Despite the potential risks, an overwhelming majority of physicians (80%) agree that copy-pasting has improved patients' hospital course documentation. An even more significant number (82%) agree that its use should continue [24]. This indicates that copy-pasting is a widely accepted and beneficial tool for medical documentation. However, healthcare providers must be aware of the potential risks of copy-pasting information into patient EMRs and take the necessary steps to ensure precision and accuracy. Alternatives to copy-pasting are available, but their use still needs to be more frequent and adequate. Singh et al. [19] revealed a correlation between copy-pasting errors and the need for urgent unplanned care, with such errors accounting for a staggering 2.6% of all mistakes. This finding emphasizes the critical need to reduce copy-pasting errors to ensure patient safety and minimize unplanned care requirements. It is clear that copy-pasting, while potentially beneficial, can harm the accuracy and reliability of data and should be used cautiously.

We must investigate the possible connection between copy-pasting and patient safety to ensure the safe use of copy-pasting; adhering to the published guidelines and protocols is essential and should be implemented, and tools and auditing features should be used to help educate and implement change. Institutes should develop policies and practice guidance to limit copy-pasting functions, and staff should be trained and educated on the proper and safe use of copy-pasting. Countercheck the impact of copy-pasting, a mechanism should be enforced to make copy-pasted material easily identifiable and traceable, succinct data display should be facilitated, and outside information should be acknowledged by reference instead of re-entering information in a note. Furthermore, EMRs should create hyperlinks between the referenced text and the referring note, author identification should be enabled, automatically entering altering entry dates should be allowed, and the origin of any copy-pasted material should be clearly stated and easily accessible. Voice recognition software and the OpenNotes initiative are valuable tools for heightening awareness of documentation accuracy. Physicians should accept ownership of the accuracy of clinical documentation and educate themselves on using documentation efficiency tools responsibly. For established patients, documentation of history and exams should be streamlined when relevant information is already contained in the medical record. Practitioners should only document changes since the last visit or pertinent items that have not changed and should refrain from repeating information that has not changed; this will help ensure the medical record is accurate and up-to-date while reducing the time spent on documentation. To ensure accuracy and safety, students should be supervised by a teaching physician when documenting services in the medical record. The teaching physician must verify all documentation or findings in the medical record, including the patient's history, physical examination, and the medical decision made by the student, and provide feedback to the student on their documentation and findings to ensure accuracy and safety. Furthermore, a system should be in place to monitor and audit the use of copy-pastng functions to ensure they are used appropriately and safely [26].

Limitation

Although this study is non-invasive, flexible, and cost-effective and provides a comprehensive understanding and grounds for further future research, it has certain definite limitations. It has limited scope, depends on current data, and lacks control and depth. It cannot be generalized. It is not an experimental randomized or prospective analysis. It is a descriptive analysis of published data with knowledge and information extracted from already published data. Descriptive research only provides a snapshot of the current situation and cannot establish cause and effect.

## Conclusions

Copy-pasting techniques are attractive as a time-saving means for busy healthcare professionals, but they increase the risk of unwanted errors in documentation. The poor and inconsiderate practice of copy-pasting can lead to medical errors, omissions, and outdated data. This may lead to patient harm, medico-legal risk, and issues related to documentation integrity. To reduce the chances of errors in patient records, healthcare professionals must be aware of these risks and ensure that all patient data registered or copied are accurate and current. Additionally, proper education and safeguards should be employed to manage copy-pasting according to some guidelines and tools. This includes providing healthcare professionals with the necessary training to understand the risks associated with copy-pasting techniques and implementing tools and processes to ensure that patient data accuracy and integrity. Healthcare organizations should consider implementing a system of checks and balances to ensure that all patient data is accurate and up-to-date. These steps, if implemented, can reduce the risk of errors in patient records and ensure accuracy.
